# Contemporary practices of Portuguese and Brazilian soccer coaches in designing and applying small-sided games

**DOI:** 10.5114/biolsport.2024.132985

**Published:** 2023-11-20

**Authors:** Filipe Manuel Clemente, José Afonso, Rui Miguel Silva, Rodrigo Aquino, Luiz Palucci Vieira, Fernando Santos, Israel Teoldo, Rafael Oliveira, Gibson Praça, Hugo Sarmento

**Affiliations:** 1 Escola Superior Desporto e Lazer, Instituto Politécnico de Viana do Castelo, Rua Escola Industrial e Comercial de Nun’Álvares, 4900-347 Viana do Castelo, Portugal; 2 Research Center in Sports Performance, Recreation, Innovation and Technology (SPRINT), 4960-320 Melgaço, Portugal; 3 Instituto de Telecomunicações, Delegação da Covilhã, Lisboa 1049-001, Portugal; 4 Faculty of Sport, Center for Research, Education, Innovation, and Intervention in Sport (CIFI2D), University of Porto, Porto, Portugal; 5 Lab Sport, Department of Sports, Centre of Physical Education and Sports, Federal University of Espírito Santo, Vitória, 29075810, Brazil; 6 Universidad César Vallejo (UCV), Facultad de Ingeniería y Arquitectura, Escuela Profesional de Ingeniería Industrial, Grupo de investigación en Tecnología aplicada a Seguridad ocupacional, Desempeño y Calidad de vida (GiTaSyC), Campus Callao, 07001 Lima, Perú; 7 Instituto Politécnico de Setúbal, Escola Superior de Educação, 2914-504 Setúbal, Portugal; 8 Life Quality Research Centre (LQRC—CIEQV, Leiria), Complexo Andaluz, Apartado, 2040-413 Rio Maior, Portugal; 9 Faculdade de Motricidade Humana, Universidade de Lisboa, 1499-002 Cruz Quebrada, Portugal; 10 Centre of Research and Studies in Soccer (NUPEF), Department of Physical Education, Universidade Federal de Viçosa, Viçosa, Brazil; 11 Sports Science School of Rio Maior–Polytechnic Institute of Santarém, 2040-413 Rio Maior, Portugal; 12 Research Center in Sport Sciences, Health Sciences and Human Development, 5001-801Vila Real, Portugal; 13 Sports Department, Universidade Federal de Minas Gerais, Belo Horizonte, Brazil; 14 Research Unit for Sport and Physical Activity (CIDAF), Faculty of Sport Sciences and Physical Education, University of Coimbra, Coimbra, Portugal

**Keywords:** Football, Surveys and questionnaires, Task constraints, Coaching, Conditioned games

## Abstract

This descriptive study aimed to investigate the current practices of Portuguese and Brazilian soccer coaches in the design and implementation of small-sided games (SSGs) in soccer. A total of 187 male coaches participated in the online survey, consisting of 82 Portuguese and 105 Brazilian individuals. These coaches held various positions within the technical staff, with 63 serving as head coaches, 38 as assistant coaches, 38 as physical trainers, and 48 in other roles related to the technical staff. Additionally, the participants represented both youth (n = 102) and adult competitive levels (n = 59), along with some who were not currently associated with a specific group. The survey consisted of 32 questions divided into three main sections: (i) the timing of SSG application, (ii) the methods used for applying SSGs, and (iii) the reasons for applying SSGs. The Chi-square test revealed a statistically significant association between nationality and the frequency of SSGs used in training sessions during the pre-season (p = 0.039) and in-season (p < 0.001). Moreover, significant association between nationality and the time allocated to employing SSGs for targeting aerobic training (p < 0.001) was found. There was a significant association between nationality and the weekly frequency of SSGs use for targeting sprint training (p = 0.019). The Chi-square test identified significant associations between nationality and the use of SSGs for targeting technical training (p = 0.002), as well as for tactical training (p = 0.002). In summary, this study underscores that SSGs are primarily employed to enhance aerobic fitness, change of direction, technical skills, and tactical behaviors. Coaches generally favor employing SSGs two to three times a week, with typical sessions lasting between 16 to 30 minutes. Notably, the major discrepancies between nationalities lie in the importance assigned to the use of SSGs. However, in practice, the formats and objectives for implementing SSGs remain relatively similar.

## INTRODUCTION

Small-sided games (SSGs) are drill-based exercises that aim to simplify the complexity of the formal game while utilizing task constraints to enhance players’ perception of specific behaviors and objectives [1]. While often referred to as SSGs, it is more accurate to describe them as “small-sided and conditioned games,” or simply “conditioned games.” This terminology better encapsulates the coach’s capacity to employ task constraints for shaping player behavior by adjusting rules and task objectives [1]. For instance, a seemingly conventional 11 vs. 11 game can undergo transformation into a conditioned game when the coach modifies the task objective (e.g., scoring in two goals instead of the regular one) or task rules (e.g., limiting players to two touches, reducing the pitch dimensions to half-field) [1,2]. However, due to the prevailing usage of SSGs in the literature as a general term, we will use “SSG” interchangeably with the more precise term “conditioned games.”

These types of games have gained popularity in soccer training environments, mainly due to their ability to exaggerate the occurrence of desired behaviors while providing an intense form of exercise [3]. As a coach, there is the potential to manipulate task objectives, such as employing small goals or focusing on ball possession as the target. Additionally, rules of play can be adjusted, encompassing variations in the number of players involved, pitch size and configuration, limitations on intra- and inter-personal coordination (e.g., restricting actions like touches on the ball or prohibiting actions like dribbling), and time constraints (e.g., setting time limits for specific actions like shooting). These adaptable features provide a versatile framework for creating dynamic and purposeful games tailored to specific training objectives, harnessing task constraints to maximize the desired outcomes. Consequently, SSGs offer a comprehensive integration of various performance dimensions, encompassing tactical-technical [2,4,5], physical/physiological [6,7], and psychological/sociological aspects [8].

Due to their flexibility, SSGs offer numerous opportunities for researchers to explore the acute and chronic adaptations resulting from manipulating different constraints [9–11]. Over the past two decades, researchers have increasingly focused on investigating the effects of these modified constraints, as SSGs provide a rich training environment that offers diverse opportunities while ensuring that players are engaged in the specific dynamics of soccer [12–14]. This emphasis on specificity enhances the effectiveness of training and optimizes the chances of improving players’ performance [6].

For instance, it is well-established that smaller formats, characterized by fewer players involved in the game, typically induce a significantly greater internal load compared to larger formats involving more players [15]. Conversely, expanding the pitch size to over 100 m^2^ substantially increases the external load demands, including parameters such as total distance covered and distance covered at higher speeds, when compared to smaller pitches [16]. Furthermore, playing with a smaller number of participants significantly increases the frequency of technical actions, such as passes, ball receptions, and dribbles [5], as well as individual tactical behaviors like penetration and delaying tactics [17]. On the other hand, larger formats typically reduce the frequency of individual technical actions while enhancing collective behaviors such as mobility and the exploration of wider areas [18].

The acute responses mentioned above can ultimately influence chronic adaptations. For example, it is well-established that small-sided games, especially those with fewer participants like 1v1 to 4v4, are particularly effective for enhancing endurance performance, making them similarly effective to running-based high-intensity interval training [19] or conventional endurance training [6]. Conversely, research indicates that when using larger formats, SSGs may not be as effective as running-based high-intensity interval training for improving sprint performance or change-of-direction, as shown in various studies [7].

Interestingly, despite the extensive research and established nature of SSGs in scientific literature, there is limited literature [20] that specifically examines how frequently sports coaches incorporate these drills into their training regimens and the factors that influence their usage. Consequently, there is a lack of systematic analysis regarding how coaches have been integrating SSGs into their daily practice. Much of the existing research has primarily focused on experimental or descriptive studies, with a specific emphasis on assessing the acute or chronic effects of SSGs on players, rather than providing insights into the actual coaching practices surrounding these games.

In terms of the frequency of SSG usage in training scenarios, a descriptive quantitative study tracked the types of exercises employed by a coach over a period of five months [21]. The results showed that, on average, coaches dedicated approximately 19.8 ± 13.4 minutes of training sessions to small-sided formats (ranging from 1v1 to 5v5), and another 19.2 ± 7.4 minutes to large-sided formats (ranging from 6v6 to 11v11) of SSGs [21].

In order to gain deeper insights into the perspectives of coaches regarding SSGs, a qualitative study published in 2017 [22] conducted interviews with two experienced soccer coaches. The findings from these interviews indicated that coaches considered SSGs important due to their ability to effectively combine tactical principles and physical fitness components within the same exercise, resulting in positive performance transfer during matches. For instance, in a qualitative report, one coach expressed, “Through the implementation of small-sided games, I can effectively implement my game model with high intensity, addressing and refining the specific situations I have in mind, all while enhancing the players’ skills.” [22] Another coach mentioned, “These games enable me to assess whether my tactical ideas are being well-executed by the players. In these drills, the athletes have the opportunity to focus on aspects that are directly applicable to the game, doing so with intensity and at a pace that closely resembles actual match conditions.” [22]

Furthermore, another qualitative study [20] focused on exploring how coaches design SSGs and align their design with their objectives in real practice scenarios. This study revealed that experienced coaches demonstrated a high level of skill in implementing task constraints that allowed for the observation of desired physiological stimuli during practice sessions [20]. Notably, there was a strong correlation (r = 0.827) between the estimated maximal heart rate of the coach and the actual maximal heart rate recorded in the players [20].

These studies [20–22] shed light on the usage and importance of SSGs in coaching practices. However, it is evident that further research is necessary to gain a comprehensive understanding of the various factors influencing the frequency and utilization of SSGs by coaches across different sports contexts. This includes considering factors related to different countries, which often have unique cultural aspects that impact training methods and approaches. Similar to studies conducted in the field of strength and conditioning [23], where practices related to physical fitness development and training load monitoring have been extensively described [24–26], a survey focusing on how coaches utilize SSGs is also required to enhance our understanding of who uses SSGs, why they are used, where they are implemented, and when they are incorporated into training sessions. This type of research has the potential to broaden our knowledge about how coaches and sports scientists perceive and utilize SSGs in their daily activities, providing valuable insights that can optimize their coaching practices.

Among various nations, the utilization of SSGs has notably thrived in Portugal and Brazil. In Portugal, the development of tactical periodization [27], alongside influential coaches like José Mourinho, has spurred the adoption of ecologically-based training exercises, leading to a significant rise in the interest and incorporation of SSGs into coaching practices. Consequently, there has been a concurrent expansion in the SSG-related literature. Brazil has closely followed this trend, as SSGs gained popularity as a foundational training approach for both youth and adult soccer players, aligning with the ecological exercise paradigm [28,29]. Given the established use of SSGs in both countries, it becomes particularly intriguing to explore and compare their practical applications within daily training sessions.

Consequently, the purpose of this study was to conduct a survey and examine the usage of SSGs in the context of soccer training. A quantitative approach was employed, utilizing questionnaires that were disseminated among coaches at various competitive levels in both Portuguese and Brazilian contexts. By collecting and analyzing this data, the study aimed to provide valuable insights into the utilization of SSGs, shed light on current practices, and offer recommendations to enhance the effectiveness of SSG implementation in soccer training programs. Additionally, the study will perform comparisons between the Portuguese and Brazilian contexts to assess how SSGs are implemented in these distinct realities.

This study offers a survey and characterization of the utilization of SSGs in daily coaching practices, employing a descriptive research design. This can prove invaluable for coaching federations and training institutions, as it provides insights into how coaches have been integrating these games into their training routines. It can also facilitate the evaluation and potential reformulation of coaching approaches, particularly if improper games have been employed in specific contexts or for certain objectives. Given its descriptive nature, the primary objective is to provide a comprehensive overview of how coaches are utilizing SSGs and to ascertain whether their use aligns with the latest scientific literature. Any identified gaps could serve as valuable feedback for coaching training centers, enabling them to enhance their educational content on this topic.

## MATERIALS AND METHODS

### Study design

This study utilized a cross-sectional study design to investigate and describe the current practices of Portuguese and Brazilian soccer coaches concerning the design and implementation of SSGs in soccer. The a priori sample size calculation utilized an effect size threshold of 0.3, which corresponds to a small effect size. This choice was made due to the absence of similar studies that could provide a mean difference between populations as a reference point. Additionally, a p-value of 0.05 and a power of 0.95 were established for the analysis. In terms of degrees of freedom, since the majority of studies involved a dichotomous questions comparing two populations (Portuguese and Brazilian), a value of 2 was assigned. Utilizing these specified parameters in the G*power software (version 3.1) for a chisquared goodness-of-fit test (option of contingency tables), the recommended a priori sample size was determined to be 172.

A diverse group of coaches, including head coaches, assistant coaches, and physical trainers, were invited to participate in the survey and share their practices and perspectives regarding the utilization of SSGs in soccer training. The primary objective was to gain a comprehensive understanding of how SSGs are currently being designed and integrated into soccer training programs, aiming to identify prevailing trends and approaches employed by coaches in both Portugal and Brazil. The study adhered to ethical standards for research involving human subjects, in accordance with the principles outlined in the Declaration of Helsinki. Prior to participating in the survey, informed consent was obtained from all participants. It is important to note that participation was entirely voluntary, and coaches had the option to withdraw from the study at any point without facing any adverse consequences or penalties.

### Setting

The online survey was administered using Google Forms and was available for participation from July 9, 2022, to July 18, 2022. The recruitment process for participants involved reaching out to coaches who expressed an interest in SSGs during a webinar held on July 9, 2022. Participants in this webinar were initially introduced to the fundamental concepts and significance of SSGs in a broad manner. This introductory session aimed to provide a comprehensive understanding of the terminologies and concepts associated with SSGs. Subsequently, the coaches received a detailed clarification regarding these games and their significance before proceeding with the survey. Additionally, the survey link was shared through the researchers’ networks, which included contacts in various soccer clubs and former students actively engaged in the sport.

To prioritize confidentiality and encourage candid responses, the survey was designed to be completely anonymous. This approach ensured that coaches could freely provide their feedback and insights in a secure and confidential manner, fostering an environment of openness and trust.

### Participants

The survey successfully gathered responses from 187 participants, all from Portugal and Brazil, and there were no missing data points. The participant breakdown included 82 Portuguese men, and 105 Brazilian men. These coaches occupied various roles within the technical staff, with 63 individuals serving as head coaches, 38 as assistant coaches, 38 as physical trainers, and 48 in other capacities related to the technical staff, such as match analysts and physiologists. Furthermore, the participants encompassed both youth (n = 102) and adult competitive levels (n = 59), with some not currently affiliated with a specific team or coaching position.

Table 1 presents an insightful overview of the participants’ characteristics and backgrounds. It includes information on their academic qualifications, certificate levels in soccer, years of experience in soccer, and their current involvement in competitive levels of the sport. This data provides valuable context for understanding the perspectives and experiences of the participants in relation to SSGs.

**TABLE 1:** Demographic characterization of survey respondents.

	Portuguese	Brazilian

	Men (N = 82)	Men (N = 105)
**Relationship with soccer**		
Soccer player or former soccer player	72 (87.8%)	57 (54.3%)

**Age**		
18–25 years old	27 (32.9%)	34 (32.4%)
26–30 years old	21 (25.6%)	16 (15.2%)
31–35 years old	13 (15.9%)	11 (10.5%)
36–40 years old	9 (11.0%)	16 (15.2%)
41–45 years old	4 (4.9%)	15 (14.3%)
46–50 years old	6 (7.3%)	4 (3.8%)
51–55 years old	1 (1.2%)	2 (1.9%)
> 55 years old	1 (1.2%)	7 (6.7%)

**Education**		
Basic education (1^st^ to 9^th^ school years)	0 (0%)	0 (0%)
Secondary school (10^th^ to 12^th^ school years)	23 (28.0%)	9 (8.6%)
Graduation	29 (35.4%)	46 (43.8%)
Post-graduation/specialization	6 (7.3%)	27 (25.7%)
Master degree	21 (25.6%)	15 (14.3%)
Ph.D. degree	3 (3.7%)	8 (7.6%)
Graduation is related with sports sciences and/or physical education	67 (81.7%)	102 (97.1%)

**Role in soccer**		
Head coach	27 (32.9%)	36 (34.3%)
Assistant coach	23 (28.0%)	15 (14.3%)
Physical trainer	16 (19.5%)	22 (21%)
Physiologist	0 (0%)	5 (4.8%)
Match analyst	3 (3.7%)	5 (4.8%)
Other	13 (15.9%)	22 (21%)

**Soccer License level**		
UEFA “C” level	26 (31.7%)	1 (1.0%)
UEFA “B” level	18 (22.0%)	0 (0%)
UEFA “A” level	5 (6.1%)	4 (3.8%)
UEFA “Pro” level	1 (1.2%)	1 (1.0%)
CBF “C” level	0 (0%)	3 (2.9%)
CBF “B” level	0 (0%)	4 (3.8%)
CBF “A” level	0 (0%)	2 (1.9%)
CBF “Pro” level	0 (0%)	0 (0%)

**Experience in soccer**		
0–5 years of experience	34 (41.5%)	52 (49.5%)
6–10 years of experience	23 (28.0%)	12 (11.4%)
11–15 years of experience	4 (4.9%)	10 (9.5%)
16–20 years of experience	4 (4.9%)	4 (3.8%)
21–25 years of experience	1 (1.2%)	3 (2.9%)
> 25 years of experience	1 (1.2%)	6 (5.7%)

**Current practice (competitive level)**		
5 to 10 years old	12 (14.6%)	12 (11.4%)
11 to 13 years old	11 (13.4%)	21 (20.0%)
14 to 16 years old	13 (15.9%)	15 (14.3%)
17 to 19 years old	8 (9.8%)	10 (9.5%)
Adults (amateurs)	15 (18.3%)	18 (17.1%)
Adults (professionals)	12 (14.6%)	14 (13.3%)

### Small-sided games survey

For this study, a comprehensive survey was developed to investigate the use and value of SSGs. The survey’s main domains, covering “when,” “how,” and “why” SSGs are used, were initially drafted by a team of three researchers, with questions based on existing literature.

To ensure the survey’s quality and validity, three independent academic experts in SSGs, recognized soccer researchers according to ExpertScape, were invited to review the draft. Their valuable feedback led to revisions and improvements in the wording and structure of the questions.

Next, the revised survey was sent to three additional experts, consisting of four soccer coaches with a minimum UEFA B license. Among them, two had no academic background in sports sciences, while the other two did. Their perspectives were considered to further enhance the survey’s content.

After incorporating feedback from both the academic experts and soccer coaches, the final version of the survey was sent back to the initial three academic experts for their final evaluation. Once their approval was obtained, the survey was presented as a supplementary file and launched online using Google Forms.

Participants were provided with a clear explanation of the survey’s purpose, research design, and assurances of anonymity, confidentiality, and data protection before proceeding. Their voluntary participation and consent were confirmed before they completed the questionnaire.

The survey encompassed a demographic questionnaire comprising 18 closed questions to gather data on the participants’ academic and professional backgrounds. In the “when” domain, 14 questions were presented to explore the frequency of Small-Sided Games (SSGs) implementation during the season, within a training week, and within individual training sessions. The “how” domain consisted of 43 questions aimed at identifying the primary objectives of using SSGs and how coaches select specific task constraints to align with these objectives. Lastly, the “why” domain featured 15 questions focused on understanding the perceived importance of utilizing SSGs for different training objectives. For the original version of the questionnaire, please refer to the supplementary file 1.

It is crucial to provide clarity regarding the terminology used in this survey. SSGs were broadly defined and explained to the participating coaches as drill-based games that maintain the dynamics of the actual game while incorporating task constraints and simplifying the format of the official game. However, it is worth noting that the more accurate term for these activities would be “conditioned games,” as it comprehensively describes their nature [1]. Nevertheless, due to the widespread use and popularity of the term “SSGs,” it was used interchangeably with the term “conditioned games” in this survey. Hence, irrespective of the particular format of play employed (e.g., 1v1 without goals, 5v5 with regular goals, 10v10 on half the pitch), all these activities were collectively categorized under the umbrella term of SSGs. This categorization was based on their shared design, which incorporates task constraints aimed at emphasizing specific behaviors and achieving desired player outcomes while preserving the fundamental dynamics of the game.

### Data acquisition and statistical procedures

The data gathered from the Google Forms survey was exported and compiled into an Excel file for analysis. For fixed response questions, a frequency analysis was conducted, which involved examining the distribution and frequency of different response options. This allowed for a quantitative assessment of the participants’ choices and preferences within the predefined response categories.

In the inferential analysis, the Chi-square test was employed to investigate potential relationships between two categorical variables, such as nationality and the survey question. When dealing with ordinal scales, the Mann-Whitney U test was utilized to assess differences between nationalities. These statistical tests were conducted using SPSS software (version 28.0.0.0, IBM, Chicago, USA), with statistical significance set at a p-value < 0.05.

## RESULTS

Table 2 presents information regarding the usage of SSGs in Portugal and Brazil, specifically focusing on the pre-season and in-season periods. No significant differences were found between the number of training sessions per week during pre-season (U = 2849.5; Z = -0.229; p = 0.819) and in-season (U = 2850.0; Z = -1.423; p = 0.155) among nationalities while using the Mann-Whitney U test.

**TABLE 2:** Characterization of the application timing (“when”) of small-sided games in soccer training during the pre-season.

	Pre-season	Pre-season	In-season	In-season

	Brazilian (n = 105)	Portuguese (n = 82)	Brazilian (n = 105)	Portuguese (n = 82)
*How many field training sessions per week does the team you manage have?*			
1	2 (2%)	0 (0%)	3 (3%)	0 (0%)
2	12 (11%)	11 (13%)	30 (29%)	10 (12%)
3	15 (14%)	19 (23%)	15 (14%)	36 (44%)
4	14 (13%)	17 (21%)	15 (14%)	16 (20%)
5	13 (12%)	18 (22%)	22 (21%)	9 (11%)
6	13 (12%)	5 (6%)	9 (9%)	4(5%)
7	4 (4%)	1 (1%)	3 (3%)	0 (0%)
> 7	9 (9%)	4 (5%)	4 (4%)	0 (0%)
Not applicable	14 (13%)	7 (9%)	14 (13%)	7 (9%)

*In how many of your weekly training sessions do you use SSGs?*			
1	5 (5%)	3 (4%)	6 (6%)	2 (2%)
2	23 (22%)	14 (17%)	24 (23%)	21 (26%)
3	14 (13%)	24 (29%)	15 (14%)	34 (41%)
4	11 (10%)	15 (18%)	19 (18%)	14 (17%)
5	14 (13%)	3 (4%)	18 (17%)	4 (5%)
6	2 (2%)	0 (0%)	5 (5%)	0 (0%)
7	1 (1%)	0 (0%)	0 (0%)	0 (0%)
> 7	0 (0%)	0 (0%)	4 (4%)	0 (0%)
All the sessions	22 (21%)	7 (9%)	0 (0%)	0 (0%)
Not applicable	13 (12%)	16 (20%)	14 (13%)	7 (9%)

*On average, how long does your training session last?*			
0–30 min	7 (7%)	1 (1%)	9 (9%)	5 (6%)
31–60 min	27 (26%)	17 (21%)	43 (41%)	22 (27%)
61–90 min	50 (48%)	53 (65%)	35 (33%)	45 (55%)
> 90 min	8 (8%)	4 (5%)	4 (4%)	3 (4%)
Not applicable	13 (12%)	7 (9%)	14 (13%)	7 (9%)

*How much time do you dedicate to the use of SSGs in your training session?*			
0–15 min	4 (4%)	3 (4%)	7 (7%)	4 (5%)
16–30 min	36 (34%)	38 (46%)	35 (33%)	35 (43%)
31–45 min	24 (23%)	20 (24%)	27 (26%)	22 (27%)
46–60 min	22 (21%)	9 (11%)	18 (17%)	10 (12%)
61–75 min	3 (3%)	4 (5%)	2 (2%)	4 (5%)
> 75 min	3 (3%)	1 (1%)	2 (2%)	0 (0%)
Not applicable	13 (12%)	7 (9%)	14 (13%)	7 (9%)

*How often do you use SSGs in your warm-up routine on a weekly basis?*			
Never	4 (4%)	5 (6%)	4 (4%)	6 (7%)
< half of the number of sessions	29 (28%)	29 (35%)	33 (31%)	35 (35%)
> half of the number of sessions	36 (34%)	33 (40%)	39 (37%)	27 (33%)
All the sessions	24 (23%)	8 (10%)	15 (14%)	7 (9%)

*How often do you integrate SSGs in the main part of your training sessions (from the end of the warm-up until the start of the cool-down)?*			
Never	2 (2%)	1 (1%)	4 (4%)	1 (1%)
< half of the number of sessions	22 (21%)	23 (28%)	25 (24%)	24 (29%)
> half of the number of sessions	48 (46%)	39 (48%)	44 (42%)	39 (48%)
All the sessions	21 (20%)	12 (15%)	17 (16%)	11 (13%)

*How often do you use SSGs during the cool-down phase on a weekly basis?*			
Never	37 (35%)	32 (39%)	35 (33%)	31 (38%)
< half of the number of sessions	26 (25%)	30 (37%)	27 (26%)	35 (43%)
> half of the number of sessions	19 (18%)	11 (13%)	23 (22%)	6 (7%)
All the sessions	9 (9%)	2 (2%)	3 (3%)	2 (2%)

The Chi-square test revealed a statistically significant association between nationality and the frequency of SSGs used in training sessions during the pre-season (X_(8)_ = 16.218; p = 0.039) and in-season (X_(7)_ = 28.165; p < 0.001). Regarding the duration of training sessions, no statistically significant association with nationality was found in the pre-season (X_(4)_ = 7.275; p = 0.122) and in-season (X_(4)_ = 8.960; p = 0.062). The Chi-square test showed no statistically significant association between nationality and the duration of SSGs per session during the pre-season (X_(6)_ = 6.220; p = 0.399) and in-season (X_(6)_ = 5.874; p = 0.437). The Chi-square test also revealed no statistically significant association between nationality and the frequency of SSG use during the warm-up phase in the pre-season (X_(4)_ = 6.832; p = 0.145) and in-season (X_(4)_ = 5.132; p = 0.274). Similarly, the Chi-square test indicated no statistically significant association between nationality and the frequency of SSG use during the main part of training in the pre-season (X_(4)_ = 2.262; p = 0.688) and in-season (X_(4)_ = 3.541; p = 0.472).

Although there were no statistically significant associations between nationality and the frequency of SSG use during the cool-down phase of training in the pre-season (X_(4)_ = 6.844; p = 0.144), significant interactions were observed in the in-season data (X_(4)_ = 12.033; p = 0.017).

Table 3 presents the frequency of answers regarding how SSGs are used for developing physical fitness. The Chi-square test did not identify any statistically significant association between nationality and the use of SSGs for targeting aerobic training (X_(1)_ = 4.475; p = 0.062). However, significant interactions were observed for sprint (X_(1)_ = 7.217; p = 0.007) and change-of-direction (X_(1)_ = 5.599; p = 0.018).

**TABLE 3:** Characterization of the application methods (“how”) of small-sided games in soccer training – improving physical fitness

	To develop aerobic fitness	To develop aerobic fitness	To develop sprint performance	To develop sprint performance	To develop change of direction	To develop change of direction

	Brazilian (n = 105)	Portuguese (n = 82)	Brazilian (n = 105)	Portuguese (n = 82)	Brazilian (n = 105)	Portuguese (n = 82)
*Do you employ SSGs as a training method focused on the development of abilities?*						
Yes	83 (79%)	59 (72%)	59 (56%)	32 (39%)	80 (76%)	54 (66%)
No	11 (10%)	17 (21%)	32 (30%)	43 (52%)	14 (13%)	23 (28%)
Not applicable	11 (10%)	6 (7%)	12 (11%)	7 (9%)	11 (10%)	5 (6%)

*If yes, what types of game formats do you most frequently use for the development of aerobic fitness?*						
1v1 to 4v4	29 (28%)	28 (34%)	41 (39%)	23 (28%)	48 (46%)	32 (39%)
5v5 to 8v8	17 (16%)	6 (7%)	9 (9%)	4 (5%)	8 (8%)	5 (6%)
8v8 to 11v11	1 (1%)	0 (0%)	0 (0%)	0 (0%)	0 (0%)	1 (1%)
Combining more than one group of formats	36 (34%)	25 (30%)	9 (9%)	5 (6%)	24 (23%)	16 (20%)

*Regarding the previous formats, do you use them most frequently?*						
Formats with numerical balance (e.g., 2v2)	21 (20%)	16 (20%)	21 (20%)	9 (11%)	20 (19%)	23 (28%)
Formats with numerical imbalance (e.g., 3v2; 2v2+2) using jokers/wildcards for whom attacking	47 (45%)	35 (43%)	32 (30%)	23 (28%)	48 (46%)	22 (27%)
Formats with numerical imbalance (e.g., 3v2; 2v2+2) using jok ers/wildcards for whom defending	15 (14%)	8 (10%)	6 (6%)	0 (0%)	12 (11%)	9 (11%)

*When do you intend to use SSGs for the development of the ability how much training session time do you allocate to this objective?*						
0–10 min	6 (6%)	10 (12%)	16 (15%)	16 (20%)	18 (17%)	12 (15%)
11–20 min	27 (26%)	34 (41%)	29 (28%)	14 (17%)	35 (33%)	30 (37%)
21–30 min	41 (39%)	10 (12%)	13 (12%)	2 (2%)	25 (24%)	11 (13%)
> 35 min	9 (9%)	5 (6%)	1 (1%)	0 (0%)	2 (2%)	1 (1%)

*How many times per week (number of sessions per week) do you intend to use SSG for the development of the ability?*						
1–2	48 (46%)	43 (52%)	40 (38%)	30 (37%)	54 (51%)	42 (51%)
3–4	33 (31%)	15 (18%)	17 (16%)	2 (2%)	25 (24%)	12 (15%)
5–7	2 (2%)	1 (1%)	2 (2%)	0 (0%)	1 (1%)	0 (0%)

Similarly, the Chi-square test did not reveal any statistically significant association between nationality and the choice of SSGs formats for targeting aerobic training (X_(3)_ = 4.329; p = 0.228), sprint training (X_(2)_ = 0.129; p = 0.938), or change-of-direction (X_(3)_ = 1.504; p = 0.681).

Furthermore, there was no statistically significant association between nationality and the numerical relationships used in SSGs for targeting aerobic training (X_(2)_ = 0.521; p = 0.771), sprint training (X_(2)_ = 4.673; p = 0.097), or change-of-direction (X_(2)_ = 5.456; p = 0.065).

However, the Chi-square test did reveal a statistically significant association between nationality and the time allocated to employing SSGs for targeting aerobic training (X_(3)_ = 18.254; p < 0.001). No significant interactions were found for sprint (X_(3)_ = 6.895; p = 0.075) and change-of-direction (X_(3)_ = 2.408; p = 0.492).

Lastly, there was no statistically significant association between nationality and the weekly frequency of SSGs use for targeting aerobic training (X_(2)_ = 3.399; p = 0.183) and change-of-direction (X_(2)_ = 2.102; p = 0.350). However, significant associations were observed for sprint training (X_(2)_ = 7.960; p = 0.019).

Table 4 provides insight into how Portuguese and Brazilian respondents use SSGs for developing technical skills and tactical behaviors. The Chi-square test identified statistically significant associations between nationality and the use of SSGs for targeting technical training (X_(1)_ = 9.748; p = 0.002), as well as for tactical training (X_(1)_ = 9.644; p = 0.002).

**TABLE 4:** Characterization of the application methods (“how”) of small-sided games in soccer training – improving technical skills and tactical behaviors

	To develop technical skills	To develop technical skills	To develop tactical behaviors	To develop tactical behaviors

	Brazilian (n = 105)	Portuguese (n = 82)	Brazilian (n = 105)	Portuguese (n = 82)
*Do you employ SSGs as a training method focused on the development of abilities?*				
Yes	90 (86%)	58 (71%)	88 (84%)	57 (70%)
No	6 (6%)	17 (21%)	6 (6%)	17 (21%)
Not applicable	9 (9%)	7 (9%)	11 (10%)	8 (10%)

*If yes, what types of game formats do you most frequently use for the development of aerobic fitness?*				
1v1 to 4v4	41 (39%)	40 (49%)	11 (10%)	8 (10%)
5v5 to 8v8	6 (6%)	1 (1%)	11 (10%)	6 (7%)
8v8 to 11v11	1 (1%)	0 (0%)	5 (5%)	0 (0%)
Combining more than one group of formats	42 (40%)	17 (21%)	61 (58%)	43 (52%)

*Regarding the previous formats, do you use them most frequently?*				
Formats with numerical balance (e.g., 2v2)	30 (29%)	34 (41%)	32 (30%)	25 (30%)
Formats with numerical imbalance (e.g., 3v2; 2v2+2) using jokers/wildcards for whom attacking	50 (48%)	19 (23%)	42 (40%)	27 (33%)
Formats with numerical imbalance (e.g., 3v2; 2v2+2) using jokers/wildcards for whom defending	10 (10%)	5 (6%)	14 (13%)	5 (6%)

*When do you intend to use SSGs for the development of the ability how much training session time do you allocate to this objective?*				
0–10 min	13 (12%)	15 (18%)	9 (9%)	7 (9%)
11–20 min	35 (33%)	33 (40%)	20 (19%)	19 (23%)
21–30 min	35 (33%)	9 (11%)	38 (36%)	25 (30%)
> 35 min	7 (7%)	1 (1%)	21 (20%)	6 (7%)

*How many times per week (number of sessions per week) do you intend to use SSG for the development of the ability?*				
1–2	50 (48%)	40 (49%)	44 (42%)	29 (35%)
3–4	32 (30%)	18 (22%)	40 (38%)	27 (33%)
5–7	8 (8%)	0 (0%)	4 (4%)	1 (1%)

Similarly, a statistically significant association was observed between nationality and the choice of SSGs formats for targeting technical training (X_(3)_ = 8.663; p = 0.034). However, no statistical interactions were found for tactical training (X_(3)_ = 3.596; p = 0.308).

Moreover, there was a statistically significant association between nationality and the numerical relationships employed in SSGs for targeting technical training (X_(2)_ = 9.363; p = 0.009). Nevertheless, no significant interactions were detected for tactical training (X_(2)_ = 1.840; p = 0.398).

Furthermore, a statistically significant association was found between nationality and the time allocated for employing SSGs in technical training (X_(3)_ = 13.791; p = 0.003). However, no significant association was observed for tactical training (X_(3)_ = 4.887; p = 0.180).

Lastly, a statistically significant association existed between nationality and the weekly frequency of SSGs use for technical training (X_(2)_ = 6.412; p = 0.041), whereas no significant association was found for tactical training (X_(2)_ = 0.814; p = 0.666).

Table 5 provides an overview of how SSGs are developed with different types of fields and task objectives, aiming to target various capacities in players. Important to disclose that penetration games involve objectives such as overcoming a line through dribbling, effectively implementing the penetration principle of play. This principle entails players facing their direct opponents, attempting to bypass them through dribbling, and advancing towards the target. The Chi-square test did not indicate any statistically significant association between nationality and pitch size for the purpose of increasing physiological stimulus (X_(1)_ = 0.906; p = 0.341), acceleration and deceleration (X_(1)_ = 0.180; p = 0.672), and developing technical actions (X_(1)_ = 0.171; p = 0.679). However, it did reveal significant associations when it came to increasing sprint (X_(1)_ = 5.136; p = 0.023) and developing tactical behaviors (X_(1)_ = 9.056; p = 0.003).

**TABLE 5:** Characterization of the task objectives used (“how”) for physiological/physical and technical/tactical stimulus

	To increase physiological stimulus	To increase physiological stimulus	To increase the running speed	To increase the running speed	To increase accelerations and decelerations

	Brazilian (n = 105)	Portuguese (n = 82)	Brazilian (n = 105)	Portuguese (n = 82)	Brazilian (n = 105)
Use bigger fields (> 1/8 of the field)	43 (41%)	28 (34%)	75 (71%)	70 (85%)	39 (37%)
Use smaller fields (< 1/8 of the field)	62 (59%)	54 (66%)	30 (29%)	12 (15%)	66 (63%)

*Which task objective do you often use?*					
Ball possession	50 (48%)	32 (39%)	13 (12%)	11 (13%)	29 (28%)
Penetration zones	29 (28%)	18 (22%)	63 (60%)	52 (63%)	26 (25%)
Small goals	15 (14%)	19 (23%)	13 (12%)	7 (9%)	28 (27%)
Youth size goals+GK	7 (7%)	9 (11%)	4 (4%)	6 (7%)	13 (12%)
Regular goals+GK	4 (4%)	4 (5%)	12 (11%)	6 (7%)	9 (9%)



	**To increase accelerations and decelerations**	**To develop technical skills**	**To develop technical skills**	**To develop tactical behaviors**	**To develop tactical behaviors**

	**Portuguese (n = 82)**	**Brazilian (n = 105)**	**Portuguese (n = 82)**	**Brazilian (n = 105)**	**Portuguese (n = 82)**
Use bigger fields (> 1/8 of the field)	28 (34%)	35 (33%)	25 (30%)	80 (76%)	76 (93%)
Use smaller fields (< 1/8 of the field)	54 (66%)	70 (67%)	57 (70%)	25 (24%)	6 (7%)

*Which task objective do you often use?*					
Ball possession	23 (28%)	55 (52%)	36 (44%)	34 (32%)	20 (24%)
Penetration zones	19 (23%)	11 (10%)	16 (20%)	15 (14%)	9 (11%)
Small goals	22 (27%)	13 (12%)	20 (24%)	6 (6%)	5 (6%)
Youth size goals+GK	7 (9%)	12 (11%)	8 (10%)	9 (9%)	10 (12%)
Regular goals+GK	11 (13%)	14 (13%)	2 (2%)	41 (39%)	38 (46%)

GK: goalkeeper

Similarly, the Chi-square test showed no statistically significant association between nationality and pitch size for increasing physiological stimulus (X_(4)_ = 4.485; p = 0.344), sprint (X_(4)_ = 2.630; p = 0.622), acceleration and deceleration (X_(4)_ = 1.698; p = 0.791), and developing tactical behaviors (X_(4)_ = 2.598; p = 0.627). However, it did reveal a significant association concerning technical development (X_(4)_ = 13.554; p = 0.009).

Table 6 illustrates the perceived relevance of using SSGs for developing different capacities. The Chi-square test indicate statistically significant association between nationality and the importance of SSGs for developing aerobic fitness (X_(4)_ = 9.689; p = 0.046), muscular power (X_(4)_ = 11.907; p = 0.018), sprint (X_(4)_ = 27.318; p < 0.001), change-of-direction (X_(4)_ = 22.642; p < 0.001), agility (X_(4)_ = 22.055; p < 0.001), offensive technical skills (X_(4)_ = 15.891; p < 0.001), defensive technical skills (X_(4)_ = 19.013; p < 0.001), offensive tactical behaviors (X_(4)_ = 12.289; p = 0.015), defensive tactical behaviors (X_(4)_ = 11.185; p = 0.025) and game model (X_(4)_ = 16.818; p = 0.002). No significant associations were found regarding SSG’s importance to maximum strength development (X_(4)_ = 2.618; p = 0.624).

**TABLE 6:** Characterization of the reasons (“why”) for using small-sided games in soccer training

	Brazilian (n = 105)	Brazilian (n = 105)	Brazilian (n = 105)	Brazilian (n = 105)	Brazilian (n = 105)

	Not Important	Slightly Important	Moderately Important	Very Important	Important
Importance of SSGs for the Development of Aerobic Fitness	2 (2%)	13 (12%)	6 (6%)	35 (33%)	49 (47%)

Importance of SSGs for the Development of Maximum Strength	9 (9%)	19 (18%)	19 (18%)	30 (29%)	28 (27%)

Importance of SSGs for the Development of Muscular Power	8 (8%)	12 (11%)	21 (20%)	32 (30%)	32 (30%)

Importance of SSGs for the Development of Maximum Running Speed (Sprint)	3 (3%)	20 (19%)	11 (10%)	30 (29%)	41 (39%)

Importance of SSGs for the Development of Change of Direction Abilities	3 (3%)	11 (10%)	9 (9%)	20 (19%)	62 (59%)

Importance of SSGs for the Development of Agility	2 (2%)	14 (13%)	4 (4%)	29 (28%)	56 (53%)

Importance of SSGs for the Development of Offensive Technical Skills (e.g., passing, receiving, shooting)	0 (0%)	10 (10%)	6 (6%)	21 (20%)	68 (65%)

Importance of SSGs for the Development of Defensive Technical Skills (e.g., tackling, intercepting)	0 (0%)	12 (11%)	3 (3%)	24 (23%)	66 (63%)

Importance of SSGs for the Development of Offensive Tactical Behaviors (e.g., penetration, offensive coverage, mobility, width)	2 (2%)	11 (10%)	5 (5%)	25 (24%)	62 (59%)

Importance of SSGs for the Development of Defensive Tactical Behaviors (e.g., defensive containment, defensive coverage, balance, concentration)	1 (1%)	10 (10%)	5 (5%)	29 (28%)	60 (57%)

Importance of SSGs for the Development of the Game Model	1 (1%)	14 (13%)	6 (6%)	29 (28%)	55 (52%)



	**Portuguese (n = 82)**	**Portuguese (n = 82)**	**Portuguese (n = 82)**	**Portuguese (n = 82)**	**Portuguese (n = 82)**

	**Not Important**	**Slightly Important**	**Moderately Important**	**Important**	**Very Important**

Importance of SSGs for the Development of Aerobic Fitness	3 (4%)	8 (10%)	7 (9%)	42 (51%)	22 (27%)

Importance of SSGs for the Development of Maximum Strength	10 (12%)	14 (17%)	19 (23%)	24 (29%)	15 (18%)

Importance of SSGs for the Development of Muscular Power	1 (1%)	11 (13%)	11 (13%)	42 (51%)	17 (21%)

Importance of SSGs for the Development of Maximum Running Speed (Sprint)	5 (6%)	12 (15%)	27 (33%)	29 (35%)	9 (11%)

Importance of SSGs for the Development of Change of Direction Abilities	0 (0%)	3 (4%)	16 (20%)	34 (41%)	29 (35%)

Importance of SSGs for the Development of Agility	0 (0%)	8 (10%)	9 (11%)	44 (54%)	21 (26%)

Importance of SSGs for the Development of Offensive Technical Skills (e.g., passing, receiving, shooting)	0 (0%)	3 (4%)	11 (13%)	33 (40%)	35 (43%)

Importance of SSGs for the Development of Defensive Technical Skills (e.g., tackling, intercepting)	1 (1%)	5 (6%)	8 (10%)	37 (45%)	31 (38%)

Importance of SSGs for the Development of Offensive Tactical Behaviors (e.g., penetration, offensive coverage, mobility, width)	0 (0%)	5 (6%)	9 (11%)	34 (41%)	34 (41%)

Importance of SSGs for the Development of Defensive Tactical Behaviors (e.g., defensive containment, defensive coverage, balance, concentration)	0 (0%)	6 (7%)	6 (7%)	40 (49%)	30 (37%)

Importance of SSGs for the Development of the Game Model	1 (1%)	6 (7%)	16 (20%)	34 (41%)	25 (30%)

## DISCUSSION

The present research aimed to characterize the usage and application of small-sided games (SSGs) in soccer by coaches from Portugal and Brazil. Out of a total sample of 201 coaches, it was found that the majority of them implement SSGs two to three times a week in both pre-season and in-season training periods. These SSG drills typically last between 16 to 30 minutes, as reported by most respondents.

Most coaches stated that they primarily incorporate SSGs during warm-up and the main part of their training sessions. Additionally, the study revealed that coaches often use SSGs to target aerobic fitness and change-of-direction, while not as frequently for sprinting. However, a significant majority reported using SSGs for stimulating technical and tactical training.

When examining the formats of SSGs, it was evident that coaches preferred smaller formats (1v1 to 4v4) for targeting aerobic fitness, change of direction, and technical skills. Conversely, medium formats (5v5 to 8v8) were more favored for developing tactical behaviors. Furthermore, coaches tended to opt for larger fields when seeking to stimulate sprinting actions and tactical training, while smaller fields were chosen for enhancing aerobic fitness, change of direction, and technical abilities.

In terms of task objectives, ball possession was the most preferred for increasing physiological stimulus, accelerations, decelerations, and technical skills. On the other hand, penetration games, where players must overcome lines for scoring, were favored for stimulating sprinting actions. Additionally, objectives using regular goals and a goalkeeper were the most frequently chosen for promoting tactical behaviors during SSGs.

### When are small-sided games employed?

The popularity of SSGs in these countries can be attributed to various factors, including the influence of tactical periodization [27,30], a well-known training approach in Portugal, which emphasizes the use of drill-based games like SSGs to enhance player development across multiple levels. As a result, it is understandable that coaches frequently integrate SSGs into their training routines, although not necessarily in every session.

The substantial use of SSGs in training sessions aligns with the findings of a previous study that examined the training process of a professional soccer team over half of a season [21]. In that study [21], it was revealed that SSGs, in various formats, accounted for up to 39 minutes of the total training session time. This parallel supports the idea that SSGs have become an integral part of modern soccer training programs, playing a crucial role in developing players’ skills.

Considering the use of SSGs during warm-up routines can be attributed to their positive effects on athletic performance, particularly in terms of reactive agility, lower limb power, and sprint performance. Previous studies [31,32] have shown that incorporating SSGs into warm-up strategies can lead to improved acute athletic responses, which can be highly beneficial for preparing players for the demands of the subsequent training session or match.

By introducing technical and tactical challenges during the warmup phase, SSGs not only enhance the physical preparedness of players but also help to stimulate decision-making abilities early on in the training session [33]. This can be especially advantageous for players as they transition from warm-up to the main part of the training session, as they are already mentally and physically primed to engage in game-like scenarios.

During the main part of the training session, which occurs after the warm-up and before the cool down, it was evident from the survey that SSGs are widely implemented by coaches from both Brazil and Portugal, regardless of the phase of the season. In fact, in more than half of the training sessions, SSGs are utilized, indicating their versatility and potential to address various training objectives.

The flexibility of SSGs allows coaches to design training sessions with specific aims in mind. These games can be employed to develop technical skills [5], hone tactical behaviors [4], or enhance physical fitness [6]. The existing literature supports the positive adaptations that can be achieved through the use of SSGs, providing a sound rationale for coaches’ inclination to incorporate them into their training routines.

The popularity of SSGs in the main part of training sessions can be attributed to their effectiveness in creating game-like scenarios [1], fostering decision-making abilities, and promoting team cohesion. The dynamic and competitive nature of these games provides an engaging and challenging environment for players, contributing to their overall development.

The widespread use of SSGs in the main part of training sessions reflects the growing recognition of their efficacy among coaches. As the body of evidence supporting the benefits of SSGs continues to grow [3,6], it is likely that coaches will continue to integrate these games into their training methodologies.

### Why are small-sided games chosen?

While Brazilian coaches largely emphasize that SSGs are crucial for developing attacking and defensive tactical principles, the majority of Portuguese coaches consider them important but not very important. Similarly, most Brazilian coaches view SSGs as very important for improving aerobic fitness and agility, whereas Portuguese coaches consider them important but not of the same high importance. These disparities in perceived importance may reflect variations in coaching philosophies, training methods, or tactical approaches in each country’s soccer culture.

Indeed, the observed differences between countries in the perceived importance of SSGs for specific components may be attributed to various factors, including differences in coaching education, available literature, or access to alternative training methods. However, it is noteworthy that SSGs are consistently considered important to very important for developing aerobic fitness, agility, technical skills, and tactical behaviors. This alignment with existing literature [6,34,35] highlights that coaches are well-informed about the advantages of using SSGs and are in line with recent evidence supporting their effectiveness.

Conversely, for improving strength, power, or sprint performance, coaches express less confidence in the utility of SSGs. This stance is also in line with existing evidence, which often suggests the superiority of other training methods for these specific physical qualities [7]. Coaches seem to recognize the need for more specialized and time-efficient approaches to target these capacities effectively, which may not be optimally addressed through SSGs alone.

### How are small-sided games implemented?

The existing literature on SSGs’ ability to improve aerobic fitness and endurance performance has been steadily growing [6], with studies suggesting that SSGs can be as effective as continuous or intermittent running-based exercises in enhancing aerobic fitness. This finding has led to the popularity of SSGs, as they enable coaches to combine technical and tactical stimulus with an appropriate aerobic workout [15]. Particularly, the smaller formats of SSGs tend to induce heart rate responses exceeding 85% of maximal heart rate [36], making them effective in providing a significant aerobic stimulus. These findings offer valuable insights into the reasons provided by coaches, with 71.3% of Portuguese and 77.2% of Brazilian coaches expressing their positive use of SSGs for developing specific physical components. The high percentage of coaches employing SSGs for this purpose aligns with the increasing body of evidence supporting the effectiveness of these drills in enhancing aerobic fitness and other physical capacities.

The coaches’ preference for using small formats of play (1v1 to 4v4) for 11 to 20 minutes with imbalance formats to stimulate aerobic fitness aligns with the existing literature on the subject. The adoption of these specific training approaches by coaches indicates their awareness of the optimal conditions required to effectively target and enhance aerobic power in their players.

Smaller formats of play, such as 1v1 to 4v4, offer several advantages for improving aerobic fitness [15]. The reduced playing area and the increased number of high-intensity actions, including sprints, accelerations, and decelerations, create a more intense and demanding physical workload for the players [37].

Additionally, the use of imbalance formats, where one team has numerical superiority over the other, further intensifies the aerobic demands on the players [38]. The team with fewer players is required to cover more ground and engage in more frequent bursts of high-intensity movements, increasing the overall metabolic demand. Moreover, the coaches’ selection of an 11 to 20-minute duration for SSGs is well-founded. Research suggests [6] that this timeframe is adequate for eliciting physiological adaptations and promoting positive changes in aerobic fitness.

It is noteworthy that coaches frequently employ smaller formats (1v1 to 4v4) of SSGs to stimulate change of direction in soccer players. The rationale behind this choice is rooted in the potential benefits of exposing players to the challenges of frequent changes of direction that occur in these game scenarios. While the heterogeneity of the number of accelerations, decelerations, and changes of direction in soccer is considerable [39], and some improvements in agility may be attributed to enhanced reaction time rather than a direct physical ability to change direction [40], studies have suggested that small-sided games can be effective forms of training to develop this skill [33].

Despite the lack of consistent research evidence specifically comparing changes of direction in small-sided games to multidirectional accelerations or sprints [35], coaches may believe that the specific demands of small formats can still contribute to the development of change-of-direction abilities in players. Indeed, agility is a multifaceted skill that encompasses perceptual-cognitive abilities, decision-making, and physical attributes. While small-sided games may not specifically target all aspects of agility, the repetitive exposure to rapid changes of direction in these game scenarios can still be beneficial for players. Coaches’ preference for using SSGs for change-of-direction training is likely based on the belief that regular exposure to such dynamic movements can lead to adaptations and improved efficiency in executing agile actions on the field.

Among the various physical qualities analyzed, coaches expressed less frequency in SSGs to improve linear sprint performance. This preference aligns with existing evidence suggesting that SSGs might not be the most efficient method for enhancing sprinting abilities in soccer players [7]. There are several reasons behind this trend observed among coaches.

Firstly, SSGs may not adequately expose players to high-intensity sprint actions, especially in smaller field formats [41]. In typical SSGs scenarios, the playing area is restricted, which may limit the distance players can cover at maximum speed [39]. As a result, players may not reach peak sprinting velocities during these games, leading to suboptimal training stimuli for enhancing linear sprint performance.

Secondly, the contextual demands of soccer matches do not always prioritize frequent sprints. Soccer involves frequent changes of pace, direction, and tactical decisions, requiring players to adopt various movement patterns. As such, players are not consistently exposed to sustained linear sprinting throughout a match, making SSGs less conducive to replicating this specific aspect of game performance [14].

The majority of coaches expressing their use of SSGs for technical skills development which is not surprising. SSGs offer a unique advantage of providing a high-intensity stimulus while seamlessly integrating technical and tactical aspects of the game. This characteristic makes them particularly attractive for coaches aiming to enhance players’ technical abilities within the context of realistic match scenarios.

Recent systematic reviews have supported this notion [34], showing that SSGs can be highly effective in improving individual technical skills, such as passing and dribbling, when compared to more analytical exercises. The dynamic and game-like nature of SSGs allows players to practice their technical skills in situations that closely resemble real-game scenarios, enhancing transferability to actual match performance.

Coaches predominantly expressed a preference for using smaller formats (1v1 to 4v4), which is consistent with the existing literature [5]. Smaller formats have been shown to increase the number of technical actions performed by each player within a given time frame [5]. This heightened frequency of actions in SSGs is likely to result in more opportunities for skill development and improvement following training interventions.

In the context of tactical behaviors, coaches also showed a significant preference for using SSGs as a means of improvement. Interestingly, in contrast to the preference for smaller formats in developing technical skills, coaches tended to opt for medium formats (5v5 to 8v8) for enhancing tactical behaviors. This choice is likely due to the larger formats allowing players to better emulate the model of play, enabling them to explore and practice important principles of the game, such as playing in unity and creating space in attack or fostering defensive concentration [4].

The analysis of task constraints and objectives employed by coaches revealed interesting trends in their preferences. Coaches tend to favor smaller fields for stimulating accelerations, decelerations, and technical skills, likely due to the increased diversity in movements and the proximity required for technical actions. On the other hand, coaches expressed a preference for using bigger fields to elicit physiological responses, promote more sprint actions, and facilitate the development of tactical behaviors.

These choices appear to align with existing evidence on the use of SSGs. Bigger fields have been shown to significantly increase players’ heart rate responses [16], making them suitable for targeting physiological fitness. Moreover, the larger playing area encourages more sprint actions, which can be crucial for specific training objectives [41]. Conversely, smaller fields may reduce the number of sprints but create a higher frequency of accelerations and decelerations [42], allowing players to practice precise technical skills.

The task objectives chosen by coaches offer valuable insights into their preferences for using specific SSG formats. Coaches primarily favored ball possession games for enhancing physiological stimulus, accelerations, decelerations, and technical actions, which aligns with the literature [43]. The emphasis on ball possession encourages players to constantly create space, leading to more frequent changes of direction and bursts of speed, as well as favoring players to circulate the ball more often, which increases the number of receives, passes, and dribbles.

For stimulating sprints, coaches mostly opted for penetration zones, which involve players overcoming a line or covering a specific distance with the ball as the primary objective of the task. This format provides opportunities for explosive bursts of speed and acceleration, contributing to the development of sprinting abilities.

In terms of tactical behaviors, the regular game format with a goalkeeper was the most favored option. This format closely replicates the conditions of a real match, encouraging players to apply tactical principles and strategies in a realistic context. The presence of a goalkeeper adds complexity to the task, requiring players to make decisions based on defensive and offensive scenarios.

### Study limitations and future research

This study has several limitations that should be acknowledged. Firstly, as a survey-based study, the responses provided by coaches are subjective in nature, and caution should be exercised while generalizing the findings. Future research could consider complementing the survey data with direct observations of coaching practices in clubs to triangulate the information.

Additionally, while the survey had a considerable number of participants, it focused solely on coaches from Portugal and Brazil. The training philosophies and coaching education programs in these countries might differ from those in other regions, limiting the generalizability of the findings to a broader international context. Therefore, caution should be exercised when applying these results to other countries or regions.

To overcome these limitations, future research endeavors should endeavor to broaden the survey’s scope to encompass coaches from diverse cultural backgrounds and geographic regions. Indeed, training approaches, models, and styles can vary significantly from one country to another. Therefore, it is essential for future research to extend the survey to encompass various regions that may employ distinct training methodologies. This will enable a more comprehensive and comparative analysis of coaching practices worldwide. The completed survey is available in supplementary file 1, facilitating its replication in different contexts and offering researchers the opportunity to compare results with our findings.

## CONCLUSIONS

This survey successfully characterized the implementation of SSGs by Portuguese and Brazilian soccer coaches, shedding light on when, how, and why these training drills are utilized. The findings indicate that coaches generally prefer to use SSGs two to three times a week, with a typical duration of 16 to 30 minutes per session. These games are predominantly employed to stimulate aerobic fitness, change of direction, as well as technical skills and tactical behaviors. Smaller formats (1v1 to 4v4) were favored for targeting various fitness, technical, and tactical capacities, while coaches adjusted task objectives and field dimensions based on the specific components they aimed to develop. Overall, the coaches considered SSGs as very important for stimulating aerobic fitness, technical skills, and tactical behaviors.

This study adds valuable insights to the existing knowledge of how coaches incorporate SSGs into their training routines. The findings may lead to future recommendations for optimizing training instructions, offer novel ideas for diversifying and modifying training practices, and provide a fresh perspective on the implementation of small-sided games in soccer training.

## Data Availability

All data generated or analyzed during this study are available at the request of the corresponding author.
